# Mesenchymal stem cells from umbilical cord matrix, adipose tissue and bone marrow exhibit different capability to suppress peripheral blood B, natural killer and T cells

**DOI:** 10.1186/scrt336

**Published:** 2013-10-15

**Authors:** Andreia Ribeiro, Paula Laranjeira, Sandrine Mendes, Isabel Velada, Cristiana Leite, Pedro Andrade, Francisco Santos, Ana Henriques, Mário Grãos, Carla M P Cardoso, António Martinho, MLuísa Pais, Cláudia Lobato da Silva, Joaquim Cabral, Hélder Trindade, Artur Paiva

**Affiliations:** 1 Blood and Transplantation Center of Coimbra, Portuguese Institute of the Blood and Transplantation, Praceta Prof. Mota Pinto, Edifíco São Jerónimo, 4.° Piso, 3001-301 Coimbra, Portugal; 2 Biocant - Technology Transfer Association, Núcleo 04, Lote 3, 3060-197 Cantanhede, Portugal; 3 Department of Bioengineering and IBB-Institute for Biotechnology and Bioengineering, Technical University of Lisbon, Avenida Rovisco Pais, 1049-001 Lisbon, Portugal; 4 Crioestaminal - Saúde e Tecnologia, S.A., Biocant Park, Núcleo 4, Lote 2, 3060-197 Cantanhede, Portugal

## Abstract

**Introduction:**

The ability to self-renew, be easily expanded *in vitro* and differentiate into different mesenchymal tissues, render mesenchymal stem cells (MSCs) an attractive therapeutic method for degenerative diseases. The subsequent discovery of their immunosuppressive ability encouraged clinical trials in graft-versus-host disease and auto-immune diseases. Despite sharing several immunophenotypic characteristics and functional capabilities, the differences between MSCs arising from different tissues are still unclear and the published data are conflicting.

**Methods:**

Here, we evaluate the influence of human MSCs derived from umbilical cord matrix (UCM), bone marrow (BM) and adipose tissue (AT), co-cultured with phytohemagglutinin (PHA)-stimulated peripheral blood mononuclear cells (MNC), on T, B and natural killer (NK) cell activation; T and B cells’ ability to acquire lymphoblast characteristics; mRNA expression of interleukin-2 (IL-2), forkhead box P3 (FoxP3), T-bet and GATA binding protein 3 (GATA3), on purified T cells, and tumor necrosis factor-alpha (TNF-α), perforin and granzyme B on purified NK cells.

**Results:**

MSCs derived from all three tissues were able to prevent CD4^+^ and CD8^+^ T cell activation and acquisition of lymphoblast characteristics and CD56^dim^ NK cell activation, wherein AT-MSCs showed a stronger inhibitory effect. Moreover, AT-MSCs blocked the T cell activation process in an earlier phase than BM- or UCM-MSCs, yielding a greater proportion of T cells in the non-activated state. Concerning B cells and CD56^bright^ NK cells, UCM-MSCs did not influence either their activation kinetics or PHA-induced lymphoblast characteristics, conversely to BM- and AT-MSCs which displayed an inhibitory effect. Besides, when co-cultured with PHA-stimulated MNC, MSCs seem to promote Treg and Th1 polarization, estimated by the increased expression of FoxP3 and T-bet mRNA within purified activated T cells, and to reduce TNF-α and perforin production by activated NK cells.

**Conclusions:**

Overall, UCM-, BM- and AT-derived MSCs hamper T cell, B cell and NK cell-mediated immune response by preventing their acquisition of lymphoblast characteristics, activation and changing the expression profile of proteins with an important role in immune function, except UCM-MSCs showed no inhibitory effect on B cells under these experimental conditions. Despite the similarities between the three types of MSCs evaluated, we detect important differences that should be taken into account when choosing the MSC source for research or therapeutic purposes.

## Introduction

Mesenchymal stem cells (MSCs) are multipotential non-hematopoietic stem cells that possess the ability to self-renew and to differentiate in response to chemical, hormonal or structural stimuli into different lineages of mesenchymal tissues, such as osteocytes, chondrocytes, neurocytes and adipocytes [1-7]. MSCs can be isolated from adult tissues, such as bone marrow, adipose tissue, endometrial polyps, menstrual blood and so on [2], and from fetal tissues, such as placenta, umbilical cord blood and matrix [8,9]. Their ability to differentiate into different tissues is variable according to their tissue of origin [4]. Bone marrow is the traditional source of human MSCs; however, there they represent a rare population of approximately 0.001% to 0.01% of total nucleated cells and their frequency tends to decline with increasing age [9-12]. Although adult MSCs have the ability to expand in culture while retaining their growth and multilineage potential [13], compared with MSCs from fetal sources, they undergo fewer cell divisions before they reach senescence [4].

All MSCs seem to share a significant number of characteristics, even if isolated from different sources: they are plastic adherent, exhibit a fibroblast-like morphology, express certain cell-surface markers (CD90, CD73 and CD105) and are distinguished from hematopoietic precursor cells and leukocytes by lacking CD34, CD45, CD14 and HLA-DR expression [3,4,14,15]. MSCs secrete several cytokines, growth factors and extracellular matrix molecules that play an important role in the regulation of hematopoiesis, angiogenesis and in immune and inflammatory response [8]. Other interesting characteristics are that MSCs can migrate and home to tissues and organs in response to growth factors, cytokines, chemokines or adhesion molecules and, therein, mediate immunomodulatory actions [10,14,16-18]. Moreover, due to their multipotency, MSC are a very attractive choice for clinical applications in several immune disorders, such as arthritis, encephalomyelitis, systemic lupus erythematosus, and in regenerative diseases, including diabetes and skin grafting [8,10,13,16,19]. Their low immunogenicity, immunomodulatory capacity and ability to differentiate into cells that regenerate damaged tissues, had already allowed the use of MSCs in clinical trials for cellular and gene therapy [10,13,14,20-22]. MSCs are able to inhibit the proliferation and function of T, B and natural killer (NK) cells, the cytolytic effects of antigen-primed cytotoxic T cells (CTL) by the induction of regulatory T cells (Treg) [14,16,20,22]. The immune modulation by MSCs seems to be mediated by secretion of soluble factors, creating an immunosuppressive microenvironment. This niche also protects MSCs from environmental insults, including cytotoxic chemotherapy and pathogenic immunity [3,23]. Beyond that, there are studies reporting that a separation of MSCs and mononuclear cells (MNC) by a semi-permeable membrane does not abrogate the inhibition of lymphocyte proliferation [20,24-28].

Different studies affirm that different molecules expressed by MSCs are responsible for, or could contribute to, suppression of lymphocyte proliferation [10,14,20,29-31]. MSCs have also been demonstrated to interfere with dendritic cells (DC) differentiation, maturation and function, by soluble factors. Consequently, this interference can be involved in suppression of T cells proliferation, as well as in the induction of regulatory antigen-presenting cells [10,18,20,30,32,33]. Moreover, MSCs seem to differently modulate the function of the various T cell subsets, which is explained in detail in the review of Duffy *et al.*[34].

In this study, we performed co-cultures of phytohemagglutinin (PHA)-stimulated MNC with MSCs from different sources (umbilical cord matrix, adipose tissue and bone marrow), for four days, to evaluate the immunomodulatory effects of MSCs on the acquisition of lymphoblast characteristics, by T and B lymphocytes, and on immune cell activation, which has been assessed by the expression of CD69, CD25 and HLA-DR on CD4^+^ and CD8^+^ T cells, B cells and NK cells. After cell sorting of the different compartments of activated T cells, we have measured transcripts for T-bet, GATA3 and FoxP3, to infer about T cell polarization to Th1, Th2 or Treg, respectively; the expression of mRNA for IL-2 was also quantified. In addition, the different compartments of activated NK cells were purified and mRNA expression of TNF-α, perforin and granzyme B was measured to evaluate the effect of MSCs on these proteins with an important role in NK cell function.

This study shows that MSCs derived from different tissues possess different immunosuppression capabilities and their action varies with the immune cell type.

## Methods

### MSCs isolation, purification and co-culture with peripheral blood mononuclear cells

Co-cultures were carried out in six-well tissue culture plates (Falcon, Becton Dickinson Biosciences, BD, San Jose, CA, USA) with peripheral blood mononuclear cells (MNC) from healthy donors and allogeneic human MSCs from the three different sources, bone marrow (BM), adipose tissue (AT) and umbilical cord matrix (UCM, also known as Wharton’s jelly MSCs), in the presence or absence of phytohemagglutinin (PHA, Irvine Scientific, Santa Ana, CA, USA), as mitogenic stimulus. Biological samples were obtained from healthy donors, with informed consent, and the study was approved by the Ethics Committees of Instituto Português de Oncologia de Lisboa Francisco Gentil (Laws n° 97/95, n° 46/2004) and Maternidade de Bissaya Barreto (ref. 356/Sec). MSCs were isolated from at least two different healthy donors for each cell source and appropriately cultured and purified to homogeneity, as previously described [35-37].

In short, MNC from BM aspirates were plated at a density of 2 × 10^5^ cells/cm^2^ on T-175 flasks (Falcon BD) in Dulbecco’s Modified Eagle Medium (DMEM) supplemented with 10% fetal bovine serum, streptomycin (0.025 μg/ml), penicillin (0.025 U/ml) (Gibco, Life Technologies, Paisley, UK), at 37°C and 5% CO_2_ in a humidified atmosphere. Medium was changed twice a week. BM-MSCs were isolated based on adherence to plastic, and near cell confluence (70 to 80%) exhausted medium was removed from the flasks, cells were washed with phosphate buffered saline (PBS, Gibco) and detached from the flask by adding Accutase solution (Sigma, St. Louis, MO, USA) for seven minutes at 37°C. Isolated BM-MSCs expressed their characteristic immunophenotype being CD73, CD90 and CD105 positive and negative for CD31, CD34, CD45 and CD80. Cell number and viability were also determined using the Trypan Blue (Gibco) exclusion method [36].

For AT-MSCs isolation, AT samples were collected into a conical tube containing PBS supplemented with 20 mg/mL of human serum albumin, washed extensively with PBS and then digested with a 0.1% (w/v) Collagenase Type II (Sigma) solution for 30 minutes at 37°C. After neutralizing the enzymatic activity of Collagenase with DMEM 10% fetal bovine serum, 1% (v/v) penicillin (10,000 U/mL)/streptomycin (10,000 g/mL) (Gibco) and 0.1% (v/v) Fungizone (Gibco), the adipose tissue was centrifuged at 2,500 rpm for 10 minutes, in order to obtain a high density stromal vascular fraction. The contaminating red blood cells were lysed by resuspending the pellet in 160 mM NH_4_Cl solution for 10 minutes at room temperature. Once again, the reaction was stopped with DMEM 10% fetal bovine serum, followed by centrifugation. Then, the suspension was filtered through a nylon mesh of 100 μm to eliminate the cellular debris and a cell count was performed. After plating and incubating overnight at 37°C, 5% CO_2_, the cells were washed with PBS in order to remove non-adherent cells. The cells were maintained at 37°C, 5% CO_2_, in a fully humidified atmosphere in DMEM 10% fetal bovine serum and the medium changed every three to four days. When cells reached near 80% confluence, cells were harvested using Accutase and plated into new T-flasks (3,000 cells/cm^2^). The number of cells and cellular viability were also determined [37].

For UCM-MSCs’ isolation, umbilical cords were rinsed with PBS (Gibco), arteries and veins were dissected and the remaining tissue fragments were digested with 0.1% collagenase type II (Sigma) for four hours at 37°C. The solution was filtered and washed with Iscove’s Modified Dulbecco’s Media (IMDM, Gibco) supplemented with streptomycin (0.025 μg/mL) and penicillin (0.025 U/mL) (Gibco). The cell number was determined using the Trypan Blue (Gibco) exclusion method. Cells were plated in T-flasks at an initial density of 10,000 cells/cm^2^ using DMEM supplemented with 10% fetal bovine serum, StemPRO® MSC Serum-Free Medium, or StemPRO® MSC SFM XenoFree (all from Gibco) culture media and kept at 37°C and 5% CO_2_ in a humidified atmosphere. When using StemPRO® MSC SFM/StemPRO® MSC SFM XenoFree cell culture, surfaces were pre-coated with CELLstartTM CTSTM (Invitrogen, Life Technologies, Paisley, UK) following the manufacturer’s instructions. After 48 hours of culture, the nonadherent cells were removed and cells were maintained by renewing the medium every three to four days. Cultures were monitored by microscopy (Olympus CK40 optical microscope, Central Valley, PA, USA) in order to assess cell morphology and spreading [35].

All assays were performed using MSCs between passage 3 and 5 and, prior to cell cultures, MSCs’ identity was confirmed by immunophenotypic analysis: 5 × 10^5^ cells were harvested to perform flow cytometry analysis for the MSCs’ markers CD105, CD73 and CD90. MNC were purified from heparinized peripheral blood by density-gradient centrifugation (Lymphoprep, Axis-Shield PoC AS, Oslo, Norway) at 1,310 × g, for 20 minutes. Cell cultures were maintained in RPMI 1640 with GlutaMax medium (Invitrogen) supplemented with 10% fetal bovine serum (Gibco) and antibiotic-antimycotic (Gibco). Control cultures consisted of MNC in the absence of MSCs, with or without PHA stimulation (17 replicates for each condition). With this strategy, different sets of co-cultures were generated: MNC + BM-MSCs and MNC + PHA + BM-MSCs (five replicates for each condition); MNC + UCM-MSCs and MNC + PHA + UCM-MSCs (seven replicates for each condition); MNC + AT-MSCs and MNC + PHA + AT-MSCs (five replicates for each condition). A total of 300,000 MNC were added to each well and, in the conditions where the cells were activated by PHA, a concentration of 10 μg/mL of the mitogen was used; in each well where MSCs were included, we added 30,000 cells, establishing a ratio of 10:1 (MNC:MSC). These plates were kept in culture for four days at 37°C, in 5% CO_2_ and 90% humidity. After four days under each condition, the cultured cells were washed with PBS 1X, pH7.4 (Gibco) and centrifuged at 540 × g for five minutes.

### Identification and quantification of the different compartments of activated T, B and NK cells

#### Immunofluorescent staining

Each cell culture was used for two purposes: (1) phenotypic study by flow cytometry, to evaluate the acquisition of lymphoblast characteristics by T and B cells and the activation kinetic of lymphocytes and (2) cell sorting and purification of the compartments of activation of lymphocytes, for gene expression analysis on T and NK cells. In order to identify different lymphocyte subtypes, we used monoclonal antibodies (mAb) conjugated with the following fluorochromes: fluorescein isothiocyanate (FITC), phycoerythrin (PE), peridinin chlorophyll protein (PerCP), phycoerythrin cyanin 7 (PE-Cy7 or PC7), allophycocyanin (APC), allophycocyanin hillite 7 (APC-H7), pacific blue (PB) and amcyan (AC). T cells were identified by CD3 PB (clone UCHT1, BD Pharmingen, San Diego, CA, USA) expression and, among this cell population, CD4^+^ T and CD8^+^ T cells were identified based on CD4 FITC (clone 13B8.2, Beckman Coulter, Brea, CA, USA) and CD8 AC (clone SK1, BD) expression, respectively; CD19 FITC (clone SJ25C1, BD) or CD19 PC7 (clone J4.119, Beckman Coulter) were used to identify B cells, and CD56 PC7 (clone N901, Beckman Coulter) positivity, in the absence of CD3 expression, for NK cells; finally, we used CD90 APC (clone 5E10, BD Pharmingen) to identify and exclude MSCs. To define the different stages of lymphocyte activation, we used mAb against CD69 PE (clone TP1.56.3, Beckman Coulter), CD25 APC-H7 (clone M-A251, BD Pharmingen) and HLA-DR PerCP (clone G46-6, BD Pharmingen).

#### Flow cytometry data acquisition and analysis

Cells were acquired on a FACS Canto™ II (BD) using FACSDiva software (BD), and 100,000 events were analyzed using Infinicyt 1.5 software (Cytognos, Salamanca, Spain). Through multi-parametric flow cytometry analysis, we identified four different subpopulations of CD4^+^ and CD8^+^ T cells: non-activated CD69^-^CD25^-^HLA-DR^-^; earlier activated CD69^+^CD25^-^HLA-DR^-^; intermediate activated CD69^+^CD25^+^HLA-DR^-^; and later activated CD69^+^CD25^+^HLA-DR^+^. In B cells, CD56^dim^ NK and CD56^bright^ NK cells, only three subpopulations were quantified: non-activated CD69^-^CD25^-^, earlier activated CD69^+^CD25^-^ and activated CD69^+^CD25^+^.

### Cell sorting of the activation compartments of T and NK cells

Cultured lymphocytes were purified by fluorescence-activated cell sorting, using FACSAria™ flow cytometer (BD). Each compartment of activated lymphocytes were sorted according to their typical phenotype: CD69^-^CD25^-^HLA-DR^-^, CD69^+^CD25^-^HLA-DR^-^, CD69^+^CD25^+^HLA-DR^-^ and CD69^+^CD25^+^HLA-DR^+^ for T cells; and CD69^-^CD25^-^, CD69^+^CD25^-^ and CD69^+^CD25^+^ for NK cells.

### Gene expression analysis

The cell sorted subsets were centrifuged for five minutes at 300 × g and the pellet resuspended in 350 μL of RLT Lysis Buffer (Qiagen, Hilden, Germany) and total RNA extraction performed with the RNeasy Mini kit (Qiagen) according to the supplier’s instructions. Total RNA was eluted in a 50 μl volume of RNase-free water. In order to quantify the amount of total RNA extracted and to verify RNA integrity, samples were analyzed using a 6000 Nano Chip kit, in an Agilent 2100 bioanalyzer (Agilent Technologies, Walbronn, Germany) and 2100 expert software, according to the manufacturer’s instructions. RNA was reverse transcribed with SuperScript III First-Strand Synthesis SuperMix for qRT-PCR (Invitrogen), according to the manufacturer’s instructions. Relative quantification of gene expression by real-time PCR was performed in the LightCycler 480 II (Roche Diagnostics, Rotkreuz, Switzerland). Real-time PCR reactions were carried out using 1 X QuantiTect SYBR Green PCR Master Mix (Qiagen), 1 X QuantiTect Primer Assay (TNF-α: QT01079561; FoxP3: QT00048286; IL2: QT00015435; Tbet: QT00042217; Gata3: QT00095501; Granzime B: QT01001875; Perforin: QT00199955) (Qiagen) and 20 ng of cDNA sample, in a total volume of 10 μl. The reactions were performed using the following thermal profile: 15 minutes at 95°C, and 40 cycles of 15 sec at 94°C, 30 sec at 55°C and 30 sec at 72°C. All samples were run in duplicate. Melting point analysis was done to ensure the amplification of the specific product. Real-time PCR results were analyzed with the LightCycler software (Roche Diagnostics). GeNorm Reference Gene Selection kit (PrimerDesign Ltd., Southampton, England) in conjunction with the geNorm software (PrimerDesign Ltd.) were used to select the reference genes to normalize data. The reference genes used for gene expression analysis, in T cells, were the splicing factor 3a, subunit 1 (SF3A1) and the topoisomerase (DNA) I (TOP1) and, in NK cells, were the ubiquitin C (UBC) and the 18S rRNA. The normalized expression levels of the genes of interest were calculated by using the delta-Ct method.

### Statistical analysis

To determine the statistical significance of the differences observed between different culture conditions, non-parametric Mann–Whitney test and Wilcoxon paired-sample test were performed, using Statistical Package for Social Sciences (IBM SPSS, version 17.0, Armonk, NY, USA). Data were expressed as mean percentage ± standard deviation. Statistically significance differences were considered when *P-*value was lower than 0.05.

## Results

### MSCs prevent T and B cells’ acquisition of lymphoblast characteristics, except for UCM-MSCs that are unable to inhibit this process in B and CD56^bright^ NK cells

**Figure 1:**
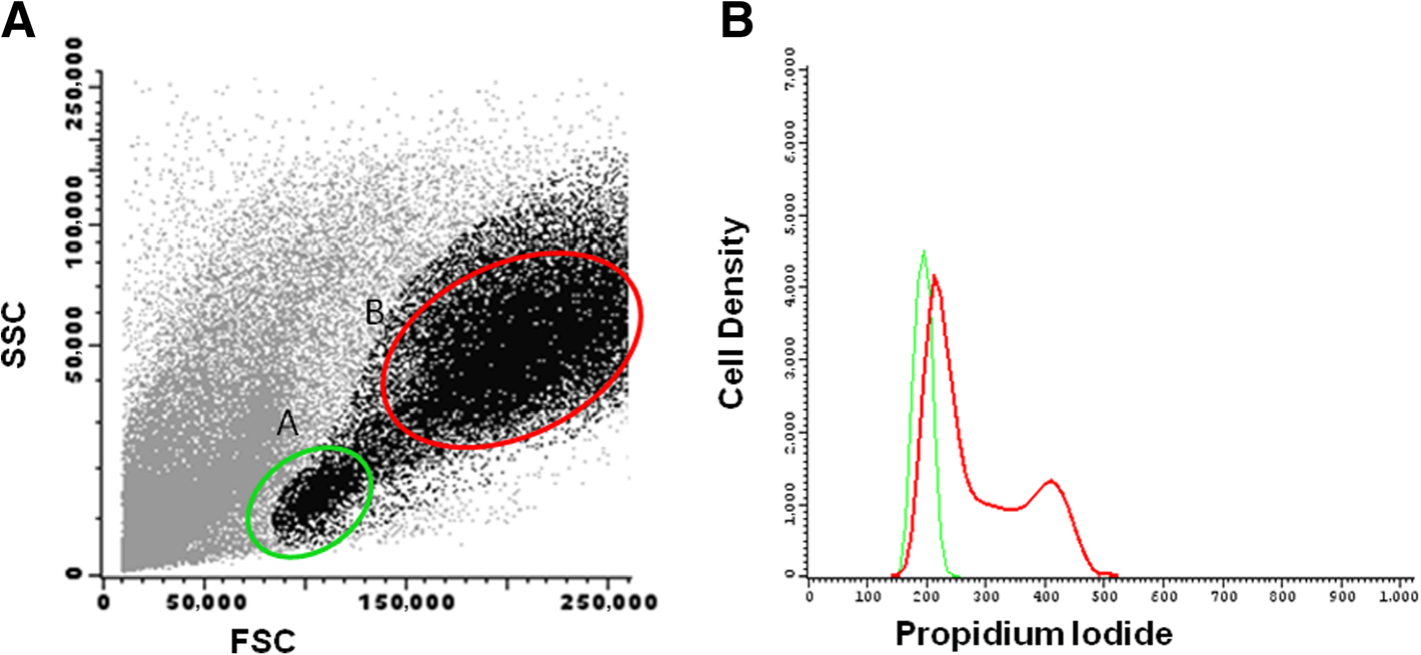
**Cells displaying increased forward scatter and side scatter light dispersion properties correspond to proliferating cells.** Left: Bivariate dot plot histogram illustrating mononuclear cell (MNC) culture after phytohemagglutinin (PHA) stimulation, displaying two distinct cell populations: **A)** MNC with low forward scatter (FSC) and side scatter (SSC) light dispersion properties and **B)** MNC with increased FSC and SSC properties. Right: Density histogram illustrating the DNA content of MNC from population A (green) and B (red), measured after propidium iodide staining, and proving that cells with increased FSC and SSC are actively proliferating.

Lymphocytes undergo multiple rounds of clonal division after mitogenic stimulation. Consequently, in the presence of PHA, lymphocytes start to proliferate and the cells acquire a lymphoblast morphology, which can be observed by flow cytometry as an increased forward scatter (FSC, corresponding to an increase in cell size) and side scatter (SSC, corresponding to an increased granularity) light dispersion properties, as shown in Figure 1. A prior analysis of FSC-Area vs FSC-Height dot plot allowed an easy identification and exclusion of doublets.

MNC were co-cultured with BM-, UCM- and AT-MSCs at the ratio of 10:1, in the presence of PHA. When compared with the MNC + PHA control assay, in the presence of MSCs, the frequency of CD4^+^ and CD8^+^ T cells with increased FSC and SSC properties was lower, as shown in Table 1 (*P* <0,05 for BM-, UCM- and AT-MSC). Although only 18% of B cells acquired lymphoblast characteristics in control assay, a marked and statistically significant decrease of the percentage of B cells with increased FSC and SSC properties occurred in the presence of BM- and AT-MSCs, whereas UCM-MSCs did not influence this process on B cells under these experimental conditions.

**Table 1:** Percentage of cells displaying increased forward scatter and side scatter light dispersion properties

**% of cells with increased FSC and SSC**	**MNC + PHA**	**MNC + PHA + BM-MSCs**	**MNC + PHA + UCM-MSCs**	**MNC + PHA + AT-MSCs**
	***n = 17***	***n = 5***	***n = 7***	***n = 5***
Total mononuclear cells	51 ± 3	31 ± 4^a; d^	41 ± 15^d^	21 ± 10^a; c; d^
Total T cells	56 ± 2	31 ± 6^a; d^	47 ± 19^d^	19 ± 13^a; c; d^
CD4^+^ T cells	54 ± 4	26 ± 3^a; d^	47 ± 22^d^	15 ± 11^a; c; d^
CD8^+^ T cells	59 ± 4	38 ± 11^a; d^	49 ± 16^d^	21 ± 7^a; b; c; d^
B cells	18 ± 3	9 ± 1^a^	18 ± 9^b^	7 ± 4^a; c; d^

Percentage (mean ± standard deviation) of cells with increased forward scatter and side scatter light dispersion characteristics after PHA stimulation, in the absence or in the presence of MSCs from different sources, within the total MNC in culture and within the following cell populations: total T cells, CD4^+^ T cells, CD8^+^ T cells and B cells.

Results are expressed as mean percentage ± standard deviation. Statistically significant differences were considered when *P* <0,05 for Mann–Whitney test:^a^comparing with MNC + PHA; ^b^comparing with MNC + PHA + BM-MSCs; ^c^comparing with MNC + PHA + UCM-MSCs. Statistically significant differences were considered when *P* <0,05 for Wilcoxon paired-sample test: ^d^comparing with MNC + PHA.

AT, adipose tissue; BM, bone marrow; MNC, mononuclear cells; MSCs, mesenchymal stem cells PHA, phytohemagglutinin; UCM, umbilical cord matrix.

### MSCs derived from different tissues influence differentially the distribution of T, B and NK lymphocytes among non-activated and activated compartments

To investigate the kinetics of lymphocyte activation, in the presence or absence of PHA and MSCs arisen from different tissues, we analyzed four subpopulations of both CD4^+^ and CD8^+^ T cells, phenotypically defined as: CD69^-^CD25^-^HLA-DR^-^ (non-activated), CD69^+^CD25^-^HLA-DR^-^ (earlier activated), CD69^+^CD25^+^HLA-DR^-^ (intermediate activated) and CD69^+^CD25^+^HLA-DR^+^ (later activated), in different culture conditions. Likewise, we identified three subpopulations of B cells, CD56^dim^ and CD56^bright^ NK cells: CD69^-^CD25^-^ (non-activated), CD69^+^CD25^-^ (earlier activated) and CD69^+^CD25^+^ (activated).

As expected, lymphocytes from MNC cultures undergo activation only after exposure to PHA (Table 2). In these culture conditions, we observe that the co-culture of MSCs from different sources presents a different effect in the inhibition of the activation.

**Table 2:** Distribution of T, B and NK cells among the respective activation compartments

		**MNC**	**MNC + BM-MSCs**	**MNC + UCM-MSCs**	**MNC + AT-MSCs**	**MNC + PHA**	**MNC + PHA + BM-MSCs**	**MNC + PHA + UCM-MSCs**	**MNC + PHA + AT-MSCs**
**Cell type**	**Subpopulation**	*n = 17*	*n = 5*	*n = 7*	*n = 5*	*n = 17*	*n = 5*	*n = 7*	*n = 5*
**Total T cells**		**82 ± 5**	**80 ± 11**	**80 ± 7**	**86 ± 5**	**86 ± 3**	**79 ± 8**	**71 ± 17**	**86 ± 5**
**CD4**^**+ **^**T cells**		**81 ± 5**	**81 ± 9**	**77 ± 9**	**84 ± 3**	**77 ± 3**	**84 ± 7**	**67 ± 8**	**76 ± 10**
	CD69^-^CD25^-^HLA-DR^-^	100 ± 0	100 ± 0	99 ± 2	100 ± 0	25 ± 7	23 ± 8	18 ± 9	43 ± 17^a; b; c; d^
	CD69^+^CD25^-^HLA-DR^-^	0 ± 0	0 ± 0	1 ± 2	0 ± 0	6 ± 1	14 ± 4^a; d^	15 ± 8^a; d^	13 ± 1^a; d^
	CD69^+^CD25^+^HLA-DR^-^	0 ± 0	0 ± 0	0 ± 0	0 ± 0	67 ± 7	58 ± 6^a^	66 ± 17	43 ± 18^a; d^
	CD69^+^CD25^+^HLA-DR^+^	0 ± 0	0 ± 0	0 ± 0	0 ± 0	3 ± 2	4 ± 2	2 ± 2	1 ± 0
**CD8**^**+ **^**T cells**		**19 ± 5**	**19 ± 9**	**23 ± 9**	**14 ± 3**	**23 ± 3**	**16 ± 7**	**33 ± 8**	**24 ± 10**
	CD69^-^CD25^-^HLA-DR^-^	99 ± 0	99 ± 2	98 ± 3	100 ± 1	18 ± 10	22 ± 3	12 ± 1^b^	41 ± 12^a; b; c; d^
	CD69^+^CD25^-^HLA-DR^-^	1 ± 0	1 ± 2	2 ± 2	0 ± 1	9 ± 1	16 ± 4^a; d^	18 ± 9^a; d^	14 ± 2^a^
	CD69^+^CD25^+^HLA-DR^-^	0 ± 0	0 ± 0	0 ± 0	0 ± 0	68 ± 11	59 ± 6^a^	67 ± 10	45 ± 14^a; b; c; d^
	CD69^+^CD25^+^HLA-DR^+^	0 ± 0	0 ± 0	0 ± 0	0 ± 0	4 ± 2	2 ± 3^d^	2 ± 2	1 ± 1
**B cell**		**2 ± 0**	**2 ± 1**	**4 ± 4**	**3 ± 2**	**3 ± 1**	**4 ± 1**	**4 ± 3**	**3 ± 2**
	CD69^-^CD25^-^	98 ± 1	95 ± 4	97 ± 3	98 ± 1	40 ± 6	63 ± 23	37 ± 9	72 ± 15^a; d^
	CD69^+^CD25^-^	2 ± 1	5 ± 4	3 ± 3	2 ± 1	56 ± 7	32 ± 15^d^	60 ± 6^b^	27 ± 14^a; d^
	CD69^+^CD25^+^	0 ± 0	0 ± 0	0 ± 0	0 ± 0	4 ± 3	5 ± 10	4 ± 4	1 ± 1
**Total NK cells**		**16 ± 5**	**19 ± 10**	**16 ± 5**	**12 ± 5**	**12 ± 2**	**20 ± 9**	**20 ± 9**	**11 ± 5**
**CD56**^**bright **^**NK cells**		**3 ± 1**	**2 ± 1**	**3 ± 2**	**4 ± 3**	**8 ± 0**	**6 ± 3**	**6 ± 5**	**8 ± 4**
	CD69^-^CD25^-^	99 ± 0	99 ± 1	99 ± 3	100 ± 1	82 ± 2	94 ± 2^a; d^	80 ± 20	97 ± 1^a; b; d^
	CD69^+^CD25^-^	1 ± 0	1 ± 1	1 ± 3	0 ± 1	17 ± 2	6 ± 2^a; d^	18 ± 17	3 ± 1^a; b; d^
	CD69^+^CD25^+^	0 ± 0	0 ± 0	0 ± 0	0 ± 0	1 ± 0	1 ± 0	2 ± 3	1 ± 0
**CD56**^**dim **^**NK cells**		**97 ± 1**	**98 ± 1**	**97 ± 2**	**96 ± 3**	**92 ± 0**	**94 ± 3**	**94 ± 3**	**92 ± 4**
	CD69^-^CD25^-^	97 ± 2	96 ± 7	97 ± 4	99 ± 1	29 ± 3	73 ± 18^a; d^	74 ± 6^a; d^	79 ± 19^a; d^
	CD69^+^CD25^-^	3 ± 2	4 ± 7	3 ± 4	1 ± 1	68 ± 2	25 ± 17^a; d^	25 ± 6^a; d^	20 ± 19^a; d^
	CD69^+^CD25^+^	0 ± 0	0 ± 0	0 ± 0	0 ± 0	3 ± 0	1 ± 1	1 ± 1	1 ± 0

Distribution (mean ± standard deviation) of total T cells, CD4^+^ and CD8^+^ T cells, B cells, total NK cells and CD56^dim^ and CD56^bright^ NK cells in the MNC culture and among the following activation stages: non-activated CD69^-^CD25^-^HLA-DR^-^, earlier activated CD69^+^CD25^-^HLA-DR^-^, intermediate activated CD69^+^CD25^+^HLA-DR^-^, and later activated CD69^+^CD25^+^HLA-DR^+^, for T cell subpopulations; and non-activated CD69^-^CD25^-^, earlier activated CD69^+^CD25^-^ and activated CD69^+^CD25^+^, for B cells and NK cell subpopulations. MNC were subjected to different culture conditions: absence or presence of PHA and absence or presence of MSCs from different sources.

Results are expressed as mean percentage ± standard deviation. Statistically significant differences were considered when *P* <0,05 for Mann–Whitney test:^a^comparing with MNC + PHA; ^b^comparing with MNC + PHA + BM-MSCs; ^c^comparing with MNC + PHA + UCM-MSCs. Statistically significant differences were considered when *P* <0,05 for Wilcoxon paired-sample test: ^d^comparing with MNC + PHA.

AT, adipose tissue; BM, bone marrow; MNC, mononuclear cells; MSCs, mesenchymal stem cells; PHA, phytohemagglutinin; UCM, umbilical cord matrix.

Analyzing CD4^+^ and CD8^+^ T cells from MNC culture, in the presence of PHA and absence of MSCs, after four days of culture, we observe that the largest proportion of cells displays an intermediate activated phenotype (Table 2); the presence of either BM- or UCM-MSCs in the cell culture increases the frequency of earlier activated cells (*P* <0.05 for both MSCs types and for both T cell subpopulations); whereas the presence of AT-MSCs is associated with a significant increase of cells within the non-activated compartment (*P* <0.05) and, to a lesser extent, in earlier activated compartment (*P* <0.05) and, consequently, is observed an important decrease in intermediate activated compartment (*P* <0.05), for both CD4^+^ and CD8^+^ T cells (Table 2).

Concerning B cells, when stimulated with PHA in the absence of MSCs, the earlier activated compartment is the most representative. As a consequence of BM and AT-MSCs, B cells are partially inhibited to proceed to the earlier activated stage; as a result, the frequency of cells in that compartment is diminished, and this decrease has statistical significance for both MSCs types (Table 2). Conversely, UCM-MSCs seem to be unable to inhibit the development of B cell activated phenotype (Table 2).

Finally, co-cultures of MSCs with MNC in the presence of PHA, present similar results for CD56^dim^ NK cells, regardless of the MSC nature and consist of an augmentation of the non-activated compartment (*P* <0.05 for all MSCs, compared with MNC + PHA), at the expense of the earlier activated compartment (Table 2). However, a distinct behavior is observed in CD56^bright^ NK cells, in which the presence of PHA has a significantly smaller effect, compared to the other lymphocyte populations considered in this study (Table 2). Despite this fact, we are able to observe a decreased percentage of CD56^bright^ NK cells undergoing activation, when co-cultured with BM- or AT-MSCs (*P* <0.05 for both); conversely, UCM-MSCs have no effect on the kinetics of CD56^bright^ NK cells’ activation (Table 2).

### MSCs differentially influence mRNA expression of FoxP3, T-bet, Gata3 and IL-2 on T cells

The analysis of gene expression levels of transcription factors and IL-2, among the different T cell activation compartments, previously sorted and purified, was performed in order to better understand the immunomodulatory mechanisms underlying MSCs.

**Figure 2:**
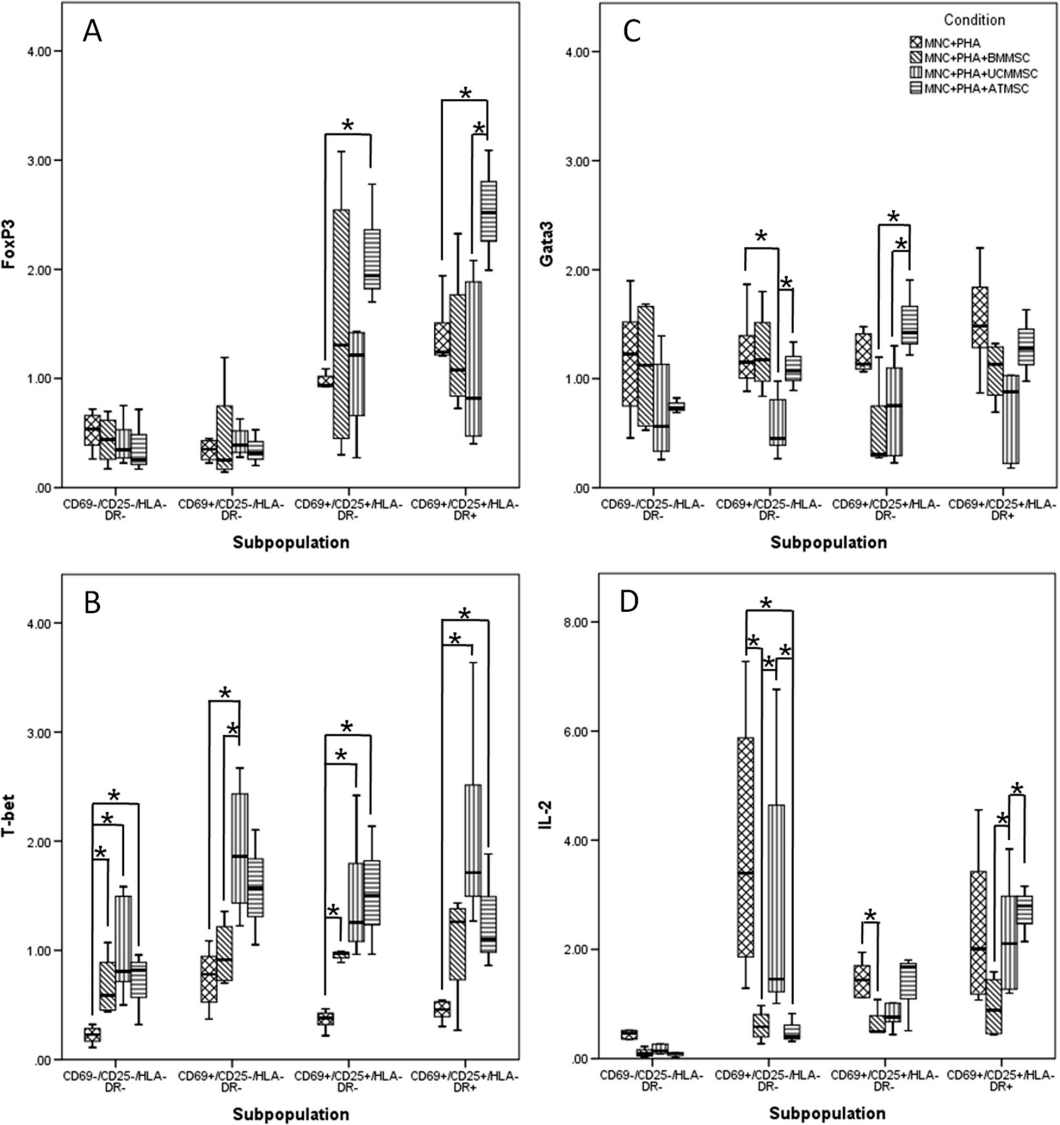
**mRNA expression of FoxP3, Tbet, GATA3 and IL-2 among the different T cells’ activation compartments.** Semi-quantitative analysis of FoxP3 **(A)**, Tbet **(B)** GATA3 **(C)**, and IL-2 **(D)** mRNA expression for each activation stage phenotypically identified on T cells: non-activated (CD69^-^CD25^-^HLA-DR^-^), earlier activated (CD69^+^CD25^-^HLA-DR^-^), intermediate activated (CD69^+^CD25^+^HLA-DR^-^) and later activated (CD69^+^CD25^+^HLA-DR^+^). * Differences statistically significant (*P* <0.05, Mann–Whitney and Wilcoxon paired-sample test). AT, adipose tissue; BM, bone marrow; MNC, mononuclear cells; MSCs, mesenchymal stem cells; PHA, phytohemagglutinin; UCM, umbilical cord matrix.

In a control assay (MNC + PHA), FoxP3 mRNA levels increase along with the progress of T cell activation; notably, the presence of AT-MSCs strongly increases FoxP3 transcripts (*P* <0.05; Figure 2A). Attending to T-bet, whose mRNA expression increases in the earlier activated stage of the control assay, an increase that is not maintained in intermediate and later activation stages, it is observed that MSCs further increase and maintain their expression along the activation process (*P* <0.05; Figure 2B). Conversely, GATA3 mRNA is not induced by PHA stimulation and the presence of MSCs do not increase GATA3 mRNA levels (Figure 2C). Finally, in control assays, the maximal expression of IL-2 mRNA is observed in an earlier activated stage, wherein the presence of MSCs of any source under study leads to a reduction, which is more notable for AT-MSCs (*P* <0.05 for BM- and AT-MSCs), as depicted in Figure 2D.

### MSCs differentially influence mRNA expression of perforin, granzyme B and TNF-α on NK cells

**Figure 3:**
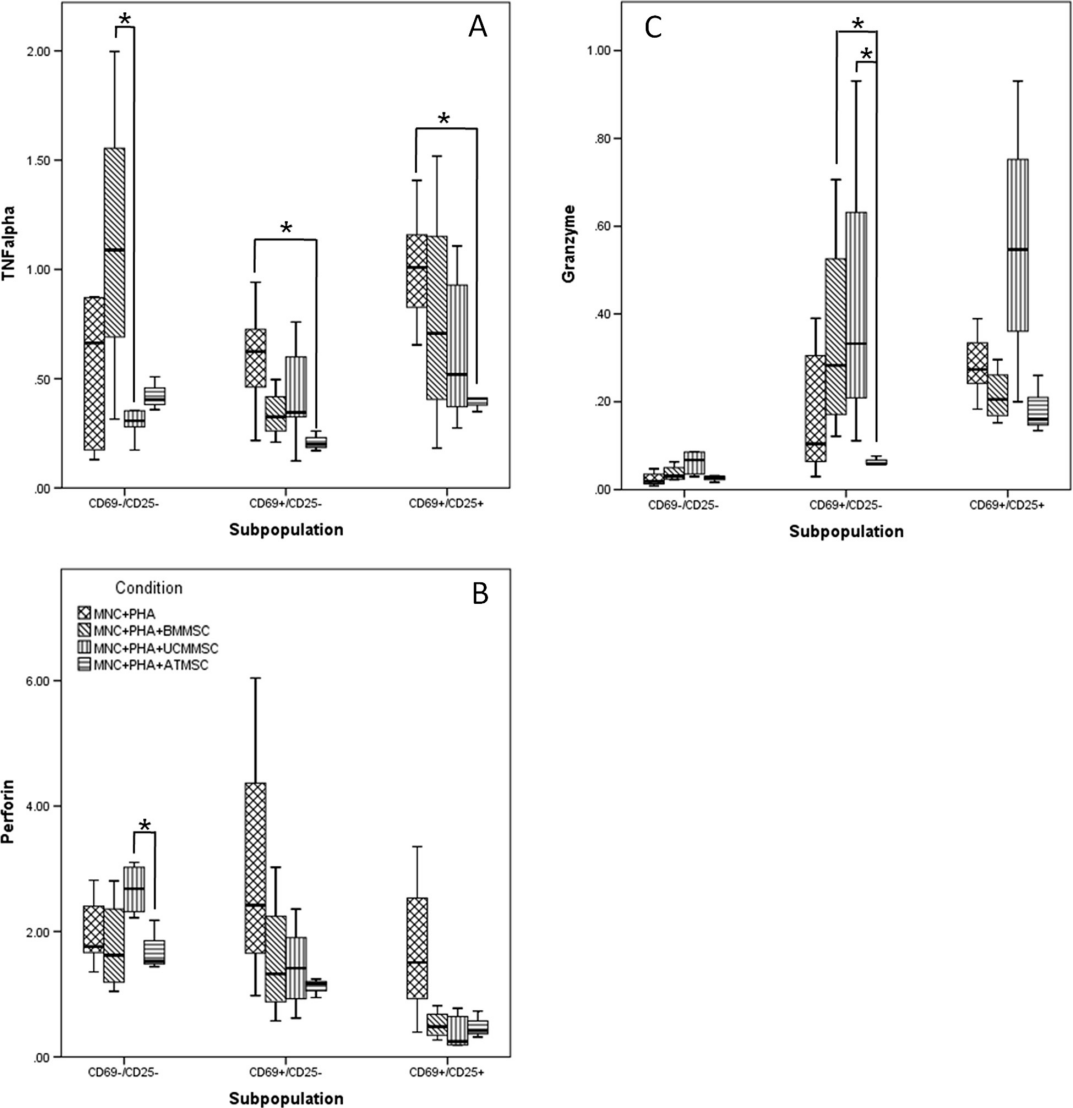
**mRNA expression of perforin, granzyme B and TNF-α among the different NK cells’ activation compartments.** Semi-quantitative analysis of TNF-α **(A)**, perforin **(B)** and granzyme B **(C)** gene expression for each cell activation stage phenotypically identified on NK cells: non-activated (CD69^-^CD25^-^), earlier activated (CD69^+^CD25^-^) and activated (CD69^+^CD25^+^). * Differences statistically significant (*P* <0.05, Mann–Whitney and Wilcoxon paired-sample test). AT, adipose tissue; BM, bone marrow; MNC, mononuclear cells; MSCs, mesenchymal stem cells; PHA, phytohemagglutinin; UCM, umbilical cord matrix.

Our data show that, on earlier activated and activated NK cells, both TNF-α and perforin mRNA expression is suppressed by all of the three types of MSCs tested (Figure 3A, B, respectively). Concerning granzyme B mRNA expression, we observed that UCM-MSCs induce an increased expression of this cytotoxic protein on activated NK cells, whereas BM- and AT-MSCs slightly reduce it (Figure 3C).

## Discussion

To assess the influence of MSCs from different sources in the induction of lymphoblast characteristics on T and B cells, ability of T, B and NK cells to progress along the activation process and mRNA expression of genes related to T cell polarization and NK cytotoxic activity, MNC cultures, in the absence/presence of PHA and in the absence/presence of MSCs, were carried out.

PHA is a lectin with the ability to bind and crosslink different cell membrane glycoproteins, leading to the polyclonal activation of lymphocytes [38]. Although TCR, CD3 and CD2 crosslinking by PHA mimics the T cells’ first signal of activation, leading to PKC activation and increasing the cytoplasmatic calcium levels, it is not sufficient to promote T cell activation, being required accessory signals given by antigen-presenting cells, which are also essential for IL-2 expression by T cells and, consequently, for their proliferation [38-41]. The early activation marker CD69 is expressed on NK, B and T cell surface 4 hours after activation and is implicated on the transcription of IL-2 and TNF-α; 12 to 24 hours after cell activation, the α subunit of IL-2 receptor (CD25) expression is up-regulated, allowing the assembling of the high-affinity IL-2 receptor on T cell plasmatic membrane; finally, between 48 to 60 hours, T cells initiate HLA-DR expression [42-45].

According to our data, both BM- and AT-MSCs, when co-cultured with PHA-stimulated MNC, inhibit B cell acquisition of lymphoblast characteristics and progression from non-activated to earlier activated stage, wherein AT-MSCs display the strongest inhibitory capability, which corroborates with the results published by Bochev *et al.*[46], who showed that AT-MSCs had a stronger ability to inhibit immunoglobulin (Ig) production by B cells than BM-MSCs. Conversely, UCM-MSCs do not influence either B cells’ ability to acquire lymphoblast characteristics or the distribution among the activation compartments.

Several studies describe the inhibitory effect of MSCs on B cell proliferation, activation, ability to differentiate to plasma cells and/or produce Ig [28,46-50], demonstrating that human BM-MSCs block stimulated B lymphocytes on G_0_/G_1_ phases of the cell cycle and interfere with the phosphorylation status of ERK1/2 and p38 MAPK [50].

Conversely to T cells, where a number of studies have been done to unveil the mechanisms beneath MSCs’ suppression of the immune response, there are few studies concerning this matter on B cells. Based on the results yielded by previous studies on T cells, it is hypothesized that programmed cell death 1 ligand 1 (PD-L1), alternatively cleaved CCL2, prostaglandin E2 (PGE2), transforming growth factor-β (TGF-β), indoleamine 2,3-dioxygenase (IDO) and hepatocyte growth factor (HGF), whose constitutive or induced expression on MSCs had already been demonstrated, might be the effector molecules supporting B cell inhibition. On mouse and murine models, PD-L1 [28,51,52] and alternatively cleaved CCL2 [52,53] were demonstrated to mediate MSCs inhibition of B cells; in contrast, TGF-β and IDO were not involved in this process [47,51]. PGE2 is known to be capable of inhibit human B cell function [54,55], but its relevance on MSC-induced B cell inhibition is still unknown.

In our experimental design, MSCs can act directly on B cells, but it should also be taken into account their suppressive effect on the other MNC in culture which, in turn, will condition B cell activation and proliferation. Indeed, by preventing T cell activation, cytokine production and down-regulating BAFF expression on DC [56], MSCs can indirectly and efficiently hamper B cell activation, proliferation and function. Likewise, there is growing evidence that the microenvironment conditions, such as the type of stimulus, presence of other cell types and MSC:B cell ratio are determinant for the effect of MSC on B cells [48,50,51,57-59]. In fact, under appropriate conditions, MSCs can enhance B cell proliferation, differentiation and Ig production [50,52,58,60].

Here we describe, for the first time, that the origin of MSCs is essential in determining B cell fate, demonstrating that AT- and BM-MSCs inhibit B cell activation and acquisition of lymphoblast characteristics, whereas UCM-MSCs do not, under the same culture conditions.

All the three types of MSCs tested, when co-cultured with MNC in the presence of PHA, inhibit the acquisition of lymphoblast characteristics by CD4^+^ and CD8^+^ T lymphocytes, more marked for AT-MSCs, followed by BM-MSCs and, finally, UCM-MSCs. Similarly, all MSCs from different tissues displayed the ability to suppress the activation of CD4^+^ and CD8^+^ T cells. Remarkably, whereas UCM- and BM-MSCs block the cell activation after the up-regulation of CD69 (at the earlier activated stage), AT-MSCs strongly inhibit the passage from non-activated to earlier activated stage, yielding a great proportion of non-activated T cells after mitogenic stimulus. Again, the effect of UCM-MSCs is milder, as the percentage of cells achieving the intermediate activated stage does not differ from that observed in the absence of MSCs. Likewise, the three types of MSCs inhibit IL-2 mRNA expression on T cells, more evident in earlier-activated stage, in which UCM-MSCs display a more moderate effect. In summary, MSCs suppress T cell immune response by reducing their proliferation and blocking their activation, what is in part a consequence of IL-2 reduction, a cytokine essential for T cell proliferation and differentiation, being described that the addition of exogenous IL-2 partially reverts MSC-mediated inhibition [61].

MSCs’ ability to inhibit T cell proliferation, activation and IL-2 expression is extensively described in the literature [59,62-68], as well as the capability to modulate the expression of other cytokines and diminish CTL cytolytic function. These capabilities are, at least in part, mediated by soluble factors produced by MSCs [63,69], namely TGF-β [70], PGE2 [59,71-73], IDO [59,74,75], leukemia inhibitory factor (LIF) [76], HLA-G5 [59,77-79], galectin [77,80-83], Jagged-1 [84], adenosine [85,86] and semaphorin-3A [80,87], and/or cellular contact and interaction between the MSCs’ surface proteins HLA-G1 [78,88], PD-L1 [28], CD200 [89,90] and B7-H4 [91-93], and the respective receptors on T cells.

T cell proliferation is inhibited by LIF [76], TGF-β [70], Jagged-1 [84], PGE2 [59,71-73], IDO [59,74,75], galectin-1 and −3 [80-83], HLA-G5 and HLA-G1 [59,77-79], PD-L1 [28], B7-H4 [91-93], semaphorin-3A [63,80,84,87] and adenosine [85]. Both semaphorin-3A and adenosine prevent T cell proliferation by antagonizing signaling pathways downstream of TCR and CD28; adenosine is also involved in the inhibition of IL-2, CD25, IFN-γ and TNFα expression and decreases cytolytic ability of CTL [85-87]. The negative co-stimulatory molecule B7-H4 prevents T cell activation, proliferation and production of IL-2 and other cytokines, by inhibiting NF-kB, ERK1/2 and JNK; B7-H4 also down-regulates CD69 and CD25 expression on T cells [91-93].

Besides the effect exerted directly on T cells, indirect suppression also occurs by inhibition of DC maturation, as demonstrated for BM-MSCs, consisting of the reduction of endocytosis and antigen processing capability, decreased expression of co-stimulatory molecules, chemokine receptors and cytokines essential for T cell activation (such as IL-12) and, consequently, diminishing DC’s capability to prime T cells, whereas stimulating IL-10 expression and, therefore, inducing tolerogenic DC’s ability to expand CD4^+^ Treg [52,57,63,72,94,95].

As previously described by our group [96] and others [97], MNC stimulation with PHA induces the expression of IFN-γ and IL-2, which activate antigen-presenting cells, contributing to the increased expression of IL-1, -6, -12 and TNF-α described on PHA-stimulated MNC [97-99], and creating the conditions for Th1 polarization (IFN-γ plus IL-12), characterized by T-bet expression. In fact, we observe an increased T-bet mRNA expression in all the three T cell activation compartments sorted and purified from MNC stimulated with PHA and the presence of MSCs seems to potentiate and preserve T-bet expression along the activation stages. When FoxP3 mRNA expression is concerned, we observe an augmentation in intermediate and later activated T cells from PHA-stimulated MNC cultures, more pronounced in the presence of MSCs, especially AT-MSCs. Thus, our data show that, using this experimental design, MSCs favor T cell differentiation toward Th1 and Treg, but we have to be aware that this effect is exerted on a small number of cells, since, in the presence of MSCs, the percentage of T cells undergoing activation is very limited.

In opposition to the results obtained for T-bet mRNA expression, MSCs support to Treg (CD4^+^FoxP3^+^) induction and expansion is widely described in the literature, contributing for this occurrence PGE2, TGF-β, HLA-G5, IDO, LIF, IL-10 and CD200, whose expression increases after MSCs exposure to pro-inflammatory factors [34,57,59,63,70,75,76,79],[89,100-106].

Although the increased T-bet expression on T cells may apparently be contradictory to previous works affirming that MSCs inhibit Th1 polarization [34,100,107-109], it is worth mentioning that, in the present work, T-bet mRNA expression was measured within each one of the four purified T cells’ activation compartments, while in the aforementioned studies the sense of T cell polarization was inferred by the pattern of cytokines expressed, specifically by the decreased IFN-γ levels. As our data clearly demonstrate, the presence of MSCs reduce the number of T cells undergoing activation, which conditions an overall reduction of IFN-γ levels on the culture supernatant, even assuming the polarization is skewed toward Th1. In fact, it has not already been described as a direct mechanism by which MSCs inhibit Th1 polarization, being hypothesized that it is an indirect effect of both the impairment of DC capability to prime naive T cells and the induction of Treg expansion [34]. Furthermore, MSCs decrease IFN-γ expression in human mixed lymphocyte reaction [63], human MNC cultures [64,81], human T cell cultures [100] and mouse differentiated Th1 cells [107,110], which will bias the conclusions of those who use IFN-γ levels to infer about Th1 polarization. Of note, recent studies yielded contradictory data, showing that, under specific conditions, MSCs may increase the levels of IFN-γ and IL-10 in the culture supernatants [64,102], which suggests that MSCs’ influence on IFN-γ expression will depend on different factors, such as the type of stimulation, type of hematopoietic cells present in cell culture and the presence of cytokines in the milieu. Finally, a recently described T-bet^+^ Th1 cell subpopulation co-expressing IFN-γ and IL-10 [111] was demonstrated to be induced and expanded by MSCs [112]. These T cells arise from Th1 polarized cells that start producing IL-10, assuming regulatory activity [112]. Likewise, TGF-β, known to be produced by MSCs, promotes IL-10 production by Th1 cells which, in turn, reduces IFN-γ expression by the same cells [113].

Thus, focusing on our experimental design, MSCs may act directly on T cells present in MNC fraction or, posteriorly, on PHA-mediated Th1-polarizing cells, since MSCs’ immunomodulatory ability increases after their contact with pro-inflammatory cytokines, whose concentration in culture medium increases after PHA-induced MNC activation. Namely, IFN-γ and/or TNF-α augment TGF-β expression by MSCs [70,104], which induces IL-10 expression by Th1 cells and, consequently, reduce their IFN-γ production. Additionally, MSCs down-regulate IFN-γR expression on T cells surface [112], rendering them less prone to respond to IFN-γ. Also, we should not discard MSCs’ effect on the DC present in the cell culture that may as well influence T cell function.

The increased mRNA expression of T-bet promoted by MSCs, observed along all the T cell activation processes and within each activation compartment, make us think that MSCs are able to suppress T cell-mediated inflammatory response by reducing the proportion of T cells that undergo activation but, once initiated the activation process, MSCs will not alter T cell differentiation’s sense in PHA-stimulated MNC, thus, T cells will continue to differentiate towards Th1. Nevertheless, as MSCs also decrease IL-2 mRNA expression on T cells undergoing activation, we may expect a limited clonal expansion of the activated lymphocytes that will further reduce the final number of effector T cells. Finally, there is evidence supporting that, after the conclusion of the differentiation process, MSCs are able to inhibit Th1 effector functions. Altogether, MSCs may inhibit Th1 immune response not by altering the sense of T cell polarization but, instead, by reducing the number of Th1 effector cells and by inhibiting the effector functions of differentiated Th1 cells.

In relation to GATA-3, although some studies confirm that MSCs modulate cytokine production by T lymphocytes toward a Th2 pattern, there is no evidence pointing to Th2 differentiation mediated by MSCs [34,108]; accordingly, our data show no influence of MSCs on GATA-3 mRNA expression. This finding is in agreement with the MSCs-mediated inhibition of B cell activation and acquisition of lymphoblast characteristics, observed in the present study.

In what concerns NK cells, PHA-induced activation is prevented for all the three types of MSCs tested, except for UCM-MSCs that are unable to inhibit the activation of CD56^bright^ NK cell subset. Once again, AT-MSCs exhibit the most pronounced inhibitory effect.

In agreement with our results, previous studies described the down-regulation of CD69 expression on NK cells under the influence of BM-MSCs [114,115]. Indeed, all the effects of MSCs already described in the literature are consistent with the inhibition of NK cell activation: MSCs prevent NK cell proliferation [59,95,116,117]; induce down-regulation of functional NK receptors [59,115-119]; inhibit the expression of pro-inflammatory cytokines (such as IFN-γ and TNF-α) [57,59,63,115-117,119] and granzyme A [119] and prevent NK cell degranulation [119]. As a result of all the effects aforementioned, MSCs hamper NK cell cytolytic activity [59,116-118]. Different evidence points to an important role of PGE2, TGF-β, IDO, HLA-G5, HLA-G1 and adenosine on NK cell inhibition, despite of some conflicting results on this matter [57,59,63,78,79,85,86,105],[115-117]. Moreover, the differences between MSCs from different sources are still unclear, with studies yielding contradictory data for BM-MSCs inhibitory ability [115,117,118].

According to our data, after PHA stimulation, NK cells express perforin and TNF-α mRNA, which is down-regulated by all three types of MSCs. This fact is in agreement with previous studies concerning TNF-α [63,116], but diverges from the results recently published by DelaRosa *et al.* showing that perforin production remains unchanged in the presence of either BM- and AT-MSCs co-cultured with purified NK cells [119]. Similarly, we observed that AT- and BM-MSCs induce a slight reduction of granzyme B mRNA expression in the later activated stage of NK cells, whereas DelaRosa *et al.* show that BM- and AT-MSCs had no effect on NK cells’ granzyme B expression [119]. It is worthwhile to remember that DelaRosa’s approach differs from ours, as in our study MSCs were co-cultured with MNC, and the presence of other immune cells in the culture may influence NK cell behavior. Finally, Yen *et al.* concluded that human embryonic stem cell-derived mesenchymal progenitors present a stronger ability to inhibit NK cell function compared to BM-MSCs, by reducing the expression of NK-activating receptors and NK cell cytotoxic activity [118]; here, we observe that both BM- and UCM-MSCs present a similar ability to inhibit CD56^dim^ NK cell activation and TNF-α and perforin mRNA expression.

In fact, it is difficult to compare results from studies concerning the MSCs’ immunomodulatory ability because MSCs are highly sensitive to the microenvironment and modulate their function according to the external conditions. Their function will vary depending on the ratio MSC:immune cells, the immune cells present in the cell culture, the immune cell activation status and the cytokines levels in the milieu [26,64,68,71,82,120], wherein pro-inflammatory cytokines, namely IFN-γ and TNF-α, increase MSCs’ expression of IDO [63,67,75], galectin-1 [82], PGE2 [63,67,70,104], CD200 [90], TGF-β [70,104], IL-10 [70] and PD-L1 [110], although there are conflicting results [121]. On the whole, this evidence point to the improvement of the immunomodulatory ability of MSCs whenever they are in an environment resembling a chronic inflammation condition. Conversely, TLR3 or TLR4 stimulation, mimicking an acute inflammation state, will reduce MSCs’ immunomodulatory ability and decrease Jagged-1 and galectin-1 expression on MSCs, similarly to that observed on Treg [63,66,82,84]. Thus, MSCs will adapt their function according to the actual conditions and requirements of the organism.

With respect to the differences observed among the different types of MSCs in the present study, the inhibitory ability of AT-MSCs is stronger than that of BM- and UCM-MSCs, which is in agreement with other published studies [67,71]. Although a few studies attempted to unravel the functional differences among AT-, BM- and UCM-MSCs [72,121,122], recent works shed light on this subject, showing that AT-MSCs express an higher level of COX-1 and PGE2 [71] and UCM-MSCs display a lower expression of PGE2 and IDO compared to BM-MSCs [67].

## Conclusion

Overall, UCM-, BM- and AT-MSCs inhibit both acquisition of lymphoblast characteristics and activation of T cells, wherein AT-MSCs display a more pronounced effect and UCM-MSCs a milder effect. Of note, AT-MSCs strongly impede T cells from proceeding from non-activated to earlier activated stages, yielding a large proportion of T cells that remain non-activated, whereas with UCM-MSCs, despite the accumulation of T cells in the earlier-activated stage, the proportion of T cells achieving the intermediate-activated state after four days of culture is equal to that observed in the absence of MSCs. Nevertheless, MSCs induce mRNA expression of the master transcriptional regulators of Treg and Th1 polarization on the T cells that proceed on the activation process. Thus, MSCs may inhibit Th1 immune response not by altering the sense of T cell polarization but, instead, by reducing the final number of Th1 effector cells (both by preventing T cells to undergo activation and by hampering IL-2 production by activated T cells) and by inhibiting the effector functions of differentiated Th1 cells.

Concerning B cells, our work showed that AT- and BM-MSCs are capable of inhibiting their activation and acquisition of lymphoblast characteristics, whereas UCM-MSCs are not. To the best of our knowledge, this is the first report showing that UCM-MSC are unable to prevent activation of B cells from MNC cultures stimulated with PHA and display a behavior completely different from their counterparts arising from other tissues, clearly showing that MSCs from different tissues may imply different immunomodulatory properties.

Finally, all three sources of MSCs tested revealed a strong inhibitory capability over CD56^dim^ NK cell activation, as well as over the production of TNF-α and perforin, whereas the activation of CD56^bright^ NK cells was only inhibited by BM- and AT-MSCs.

## Abbreviations

AC: Amcyan; APC: Allophycocyanin; APC-H7: Allophycocyanin hillite 7; AT: Adipose tissue; BAFF: B-cell activating factor; BCR: B cell receptor; BM: Bone marrow; CTL: Cytotoxic T lymphocyte; DC: Dendritic cells; ERK: Extracellular signal-regulated kinase; FITC: Fluorescein isothiocyanate; FoxP3: Forkhead box P3; FSC: Forward scatter; GATA3: GATA binding protein 3; HGF: Hepatocyte growth factor; HLA: Human leukocyte antigen; IDO: Indoleamine 2,3-dioxygenase; IFN-γ: Interferon-γ; Ig: Immunoglobulin; IL: Interleukin; JNK: Jun N-terminal kinase; LIF: Leukemia inhibitory factor; LPS: Lipopolysaccharide; mAb: Monoclonal antibody; MAPK: Mitogen-activated protein kinase; MNC: Mononuclear cells; MSCs: Mesenchymal stem cells; NF-kB: Nuclear factor-kappa B; NK: Natural killer; PB: Pacific blue; PBS: phosphate-buffered saline; PD-L1: Programmed cell death 1 ligand 1; PE: Phycoerythrin; PE-Cy7 or PC7: Phycoerythrin cyanin 7; PerCP: Peridinin chlorophyll protein; PGE2: Prostaglandin E2; PHA: Phytohemagglutinin; PKC: Protein kinase C; SSC: Side scatter; STAT3: Signal transducer and activator of transcription 3; TCR: T cell receptor; TGF-β: Transforming growth factor-β; TLR: Toll like receptor; TNF-α: Tumor necrosis factor-α; Treg: Regulatory T cells; UCM: Umbilical cord matrix.

## Competing interests

The authors declare they have no competing interests.

## Authors’ contributions

AR was responsible for manuscript drafting, cell sorting, cell culture experiments, and immunophenotypic studies. PL performed manuscript drafting, data analysis and interpretation. SM was responsible for cell culture experiments, cell sorting and immunophentypic studies. IV carried out gene expression studies. CL, PA, and FS performed mesenchymal stem cell isolation and expansion. AH performed cell sorting and immunophenotypic studies. MG contributed to mesenchymal stem cell isolation and expansion and manuscript revision. CC contributed to study design, mesenchymal stem cell isolation and expansion, and manuscript revision. AM contributed to gene expression studies and manuscript revision. MLP, JC and HT contributed to the study design, and manuscript revision. CLS contributed to study design, mesenchymal stem cells isolation and expansion, and manuscript revision. AP conceived the study, and contributed to study design and coordination, analysis and interpretation of data, and manuscript revision. All authors read and approved the final manuscript.
